# Report of Diseases, from August 10th to November 10th, 1822; Being a List of the Complaints Affecting Adults, Treated at the Westminster General Dispensary

**Published:** 1822-12

**Authors:** R. Macleod

**Affiliations:** One of the Physicians to That Institution.


### STATISTICAL MEDICINE.

ART. I.—*Report of Diseases, from August 10th to November 10th, 1822; being a List of the Complaints affecting* ADULTS, *treated at the Westminster General Dispensary.* By R. MACLEOD, M.D. one of the Physicians to that Institution.

***Diseases affecting particular Organs.***

| | | | | | |
|---|---|---|---|---|---|
| Head and Nervous System | | Apoplexy (decided) | | 2 | |
| | | ——— (threatened) | | 9 | |
| | | Paralysis (Hemiplegia) | | 1 | |
| | | Epilepsy | | 1 | |
| | | Hysteria | | 10 | |
| | | Chorea | | 1 | |
| | | Tremors | | 1 | =25 |
| Particular Nerves | | Neuralgia (of the Supraorbitary Nerve) | | | 1 |
| Nostrils, Fauces, and Throat | | Catarrh | | 5 | |
| | | Cynanche | Tonsillaris | 7 | |
| | | | Laryngea | 1 | |
| | | | Parotidea | 1 | |
| | | Epistaxis | | 1 | =15 |
| Organs of Respiration | | Dyspnœa (cause not apparent) | | 3 | |
| | | Bronchitis | Acute | 3 | |
| | | | Chronic | 19 | |
| | | Pneumonia | | 6 | |
| | | Hæmoptisis | | 3 | |
| | | Phthisis | | 20 | =54 |
| Organs of Circulation | | Palpitation | | 1 | |
| | | Angina Pectoris | | 2 | = 3 |
| Organs of Digestion | Stomach | Dyspepsia (Simple) | | 30 | |
| | | ——— with Gastrodynia | | 3 | |
| | | ——— with Pyrosis | | 1 | |
| | | ——— with Hæmatemesis | | 1 | |
| | Bowels | Constipation (Simple) | | 3 | |
| | | ——— with Colic | | 4 | |
| | | Enteritis | | 1 | |
| | | Diarrhœa | Simple | 3 | |
| | | | with Blood | 2 | |
| | Liver | Hepatitis, Acute | | 1 | |
| | | ———, Chronic | | 8 | |
| | | Icterus | | 2 | |
| | All three | Cholera | | 1 | =60 |
| Organs of Urine | | Nephralgia | | 1 | |
| | | Dysuria | | 2 | |
| | | Palsy of the Bladder | | 1 | = 4 |
| Organs of Generation | | Amenorrhœa | | 2 | |
| | | Menorrhagia | | 4 | |
| | | Leucorrhœa | | 4 | =10 |
| Skin—Eruptions | Acute | Erysipelas | | 1 | |
| | | Scarlatina | | 1 | |
| | | Variola | | 1 | = 3 |
| | Chronic | Lichen | | 1 | |
| | | Lepra | | 1 | |
| | | Porrigo | | 2 | |
| | | Papulæ | | 1 | = 5 |

***Diseases affecting various Parts.***

| | | | | |
|---|---|---|---|---|
| Muscles, Tendons, Joints, &c. | Gout | | 2 | |
| | Rheumatism | Acute | 8 | |
| | | Chronic | 22 | =32 |

*Diseases not easily referred to particular Organs.*

| | | | |
|---|---|---|---|
| Fevers | Continued | | 17 |
| Dropsies | Anasarca | 1 | |
| | Ascites | 5 | |
| | Hydrothorax | 2 | = 8 |
| Names of Diseases not mentioned | | | 10 |
| | Total number of Adult Patients | | *247 |

Fatal Cases, 4.—Diseases: Palsy of the Bladder, 1; Hæmoptisis, 1; Fever, 1; Phthisis, 1.

Any one, in looking over the above table, may naturally be astonished at seeing so many formidable diseases, and so few deaths; and it must at once be obvious that these cannot be regarded as indicating the rate of mortality. There are, for example, in the list no fewer than twenty cases of pulmonary consumption, and only one death from that disease: this is accounted for by the nature of the Institution, from the records of which the report is made. During the three months included in this account, twenty patients have been admitted labouring under the various stages of phthisis: of these, about ten are still in attendance, most of them gradually getting worse as winter approaches; five or six have got a little better, and been discharged; and the remainder have discontinued their visits, either from death or inability to come. Patients in consumption frequently attend till within a few days of their dissolution, when they suddenly disappear, and, either from living without the visiting district of the Dispensary, or other causes, are heard of no more. We are sorry not to be able to report any as cured; for, although we have tried all the various remedies recommended by those who place more confidence in the powers of medicine than we are able to do, yet it has never fallen to our lot to witness a case of *genuine* phthisis *cured.* By putting cured in italics, we mean that we have seen a few instances, which appeared to us to be tubercular phthisis, in which the patients have recovered; yet this result never seemed to follow from the administration of medicines supposed to exert particular influence on pulmonary complaints, as antimony, digitalis, or prussic acid; but, on the contrary, from the general system appearing to recruit under the use of light nourishing diet, and such tonics as have not increased the fever,—particularly iron. We beg not to be misunderstood: we are fully aware that an opposite mode of treatment is requisite in the commencement of the disease; but even here we suspect that depletion, particularly venesection, is often employed too freely; and we do most firmly believe that the system of bleeding a consumptive patient every time the pain becomes more acute, tends very much to hasten his death.

Seventeen cases of fever are marked in the list; of these, almost the whole have been admitted during the last six weeks. The disease, however, has been of an unusually mild type,—so much so, indeed, that many patients, in whom it has nevertheless been well marked, have not been confined to bed. When it becomes more severe, this proceeds

* This number includes only the adults treated by Dr. MACLEOD, exclusive of the other medical officers; the total number of patients during the three months exceeding 1200.

rather from debility than any marked determination to particular organs, or inflammatory action of the general system. In one patient, there was acute pain in the chest, putting on the appearance of pleuritis,—this was the only instance in which I had recourse to bleeding, (twelve ounces;) and the only instance in which the patient *died.* The treatment has rather consisted in avoiding any thing likely to do harm, than in any active attempts to do good: in short, it has been "la pratique expectante."

In the preceding report, (see the Number of this Journal for September,) I mentioned a case of tic douloureux, which got well under the use of the subcarbonate of iron: another will be seen in the above list, but which has resisted the iron in doses of four scruples three times a-day, and sulphate of quinine in doses of sixteen grains a-day; it is still under treatment.

Of the four cases which proved fatal, one only was examined after death: it is reported as Hemoptisis, taking the name from the prominent symptoms,—a plan which it becomes necessary to adopt at an extensive charity, where the object is more to relieve the complaints than find out their exact designation in the nosology. The patient was a strong muscular man, about fifty, who had been affected for some months with oppressed respiration and frequent cough, but without considerable pain; nor did the pulse indicate inflammatory action. He frequently evacuated very dark bloody fluid, which appeared, from the oppression of the breath preceding it, and the cough by which it was brought up, to come from the lungs, although the colour was more that of blood from the stomach. The principal remedies employed were venesection and superacetate of lead, but without benefit; and he died with much laborious breathing, being very little reduced in muscular power. On opening the body, the only appearances of disease were in the chest: there was effusion of bloody serum in the left cavity, to the extent of about half a pint, and some very slight adhesions on the same side between the pleura costalis and pulmonalis. The right lung seemed, externally, very dark,—nearly black; it felt rather more dense than usual, but not considerably so. On cutting into it, it seemed throughout pervious to air. On a superficial view externally, or of the section of its internal structure, the dark colour appeared universal; but more minute inspection proved this not to be the case: the black part was disseminated in very close points, or stars, through structure that appeared healthy. On squeezing a portion, a blackish fluid, like treacle, was forced out; which, as well as the lung, in a bright light, verged to green. This peculiar structure pervaded the whole of the right lung, and part of the two upper lobes of the left: it was pretty uniform. The back parts seemed to communicate with the air-tubes; for fluid, of a similar appearance, was easily forced into them; but there was nothing like vomica or abscess in any part. This constitutes the best example I have seen of the third variety of *melanosis,* described by M. Laennec.

